# Hypoxia in Diabetic Kidneys

**DOI:** 10.1155/2014/837421

**Published:** 2014-06-23

**Authors:** Yumi Takiyama, Masakazu Haneda

**Affiliations:** Division of Metabolism and Biosystemic Science, Department of Medicine, Asahikawa Medical University, Midorigaoka Higashi 2-1-1-1, Asahikawa 078-8510, Japan

## Abstract

Diabetic nephropathy (DN) is now a leading cause of end-stage renal disease. In addition, DN accounts for the increased mortality in type 1 and type 2 diabetes, and then patients without DN achieve long-term survival compatible with general population. Hypoxia represents an early event in the development and progression of DN, and hypoxia-inducible factor- (HIF-) 1 mediates the metabolic responses to renal hypoxia. Diabetes induces the “fraternal twins” of hypoxia, that is, pseudohypoxia and hypoxia. The kidneys are susceptible to hyperoxia because they accept 20% of the cardiac output. Therefore, the kidneys have specific vasculature to avoid hyperoxia, that is, AV oxygen shunting. The NAD-dependent histone deacetylases (HDACs) sirtuins are seven mammalian proteins, SIRTs 1–7, which are known to modulate longevity and metabolism. Recent studies demonstrated that some isoforms of sirtuins inhibit the activation of HIF by deacetylation or noncatalyzing effects. The kidneys, which have a vascular system that protects them against hyperoxia, unfortunately experience extraordinary hypernutrition today. Then, an unexpected overload of glucose augments the oxygen consumption, which ironically results in hypoxia. This review highlights the primary role of HIF in diabetic kidneys for the metabolic adaptation to diabetes-induced hypoxia.

## 1. Hypoxia in Diabetic Kidney

Diabetic nephropathy (DN) is now a leading cause of end-stage renal disease (ESRD) and therefore constitutes a major factor in progressive kidney disease. In addition, in absence of DN, diabetes is not associated with a large increase in mortality risk in type 1 or type 2 diabetes [1, 2]. Chronic hypoxia and tubulointerstitial fibrosis are presently considered to be a common pathway for various progressive kidney diseases including DN, and hypoxia inducible factor- (HIF-) 1*α* plays an important role in these pathological mechanisms [3–6].

Earlier studies have demonstrated that hypoxia represents an early event in the development and progression of experimental DN [7, 8] and an increased HIF-1*α* expression in diabetic kidneys compared to the kidneys of control rats [9] and normal human kidneys [10]. Intrarenal oxygenation can be assessed noninvasively in subjects with type 1 or type 2 diabetes by blood oxygenation level dependent magnetic resonance imaging (BOLD MRI) [11–13]. Inoue et al., especially, demonstrated hypoxia in the renal parenchyma of chronic kidney disease (CKD) patients with or without diabetes, using diffusion-weighted (DW) MRI and BOLDMRI [13], and confirmed intrarenal hypoxia in patients with diabetes, suggesting that factors other than tubulointerstitial alteration (such as loss of peritubular capillaries) determine the degree of hypoxia in the renal cortex [13]. The kidneys are less than 1% of total body weight and use 10% of total oxygen consumption. Na^+^/K^+^-ATPase in cortical proximal tubular cells consumes 80% of the oxygen for kidneys [14], and kidney needs 3%–18% of the total oxygen consumption for its basal metabolism [15].

Usually, tissue oxygen tension is determined by the balance between the blood flow and tissue oxygen consumption. However, the kidneys do not fall under such a simple calculation, because the kidneys receive blood to regulate the blood volume and composition, not for their own benefit. Increased renal blood flow increases the glomerular filtration rate, which in turn increases the rate of sodium reabsorption, which increases the tissue oxygen consumption.

Additionally, diabetes induces glomerular hyperfiltration [16] and increases tubular sodium and glucose reabsorption through sodium glucose cotransporters (SGLTs), which enhances Na^+^/K^+^-ATPase activity, resulting in an increased ouabain-sensitive oxygen consumption [17]. Therefore, in diabetic kidney, hyperfiltration triggers a vicious circle between increasing oxygen delivery and increasing oxygen consumption that leads to more needs for oxygen supply.

Previous studies have shown that the loop diuretic furosemide, which inhibits reabsorption in the medullary thick ascending limb, increased medullary oxygen tension by reducing oxygen consumption rather than by increasing medullary blood flow [18, 19]. Intriguingly, the novel antidiabetic drug and SGLT2 inhibitor empagliflozin attenuate renal hyperfiltration in subjects with type 1 diabetes [20], suggesting the potential role of SGLT2 inhibitors in renoprotection in diabetic kidneys by inhibiting proximal tubular oxygen consumption.

In an in vitro study, high glucose failed to induce or enhance the expression of HIF-1*α* protein in human renal proximal tubular cells (HRPTECs) in normoxia or in hypoxia, respectively [21]. In addition, it was found in the same study that high glucose abolished the Pasteur effects, which are adaptive responses to hypoxic stress by decreased oxidative phosphorylation and an increase in anaerobic fermentation, and that high glucose decreased mitochondrial efficiency by uncoupling oxygen consumption from ATP production [21]. Furthermore, in another cultured system, high glucose blunted the vascular endothelial growth factor (VEGF) response to hypoxia in immortalized rat proximal tubular cells via the oxidative stress-regulated HIF/hypoxia-responsible element (HRE) pathway [22]. Collectively, hyperglycemia itself fails to enhance HIF-1 expression, whereas it attenuates HIF-1 mediated responses to hypoxia. Thus, hyperglycemia induces hypoxia and hypoxia-induced HIF-1 expression in the diabetic kidneys through hemodynamic or/and metabolic changes in vivo, not by a direct effect.

It is well known that there are some pathways that are activated by hyperglycemia, such as the polyol pathway, the hexosamine pathway, the protein kinase C activation, and the AGE pathway [23]. However, every therapeutic challenge using the inhibitors for these pathways failed to cure diabetic complications including DN, indicating the inappropriate therapeutic strategy and needs for an alternative approach. Recently, Friederich-Persson et al. studied the effects of the mitochondrial uncoupler dinitrophenol (DNP) in rats [24]. DNP did not affect renal blood flow, glomerular filtration, blood glucose, or oxidative stress but increased the kidney oxygen consumption and resulted in intrarenal tissue hypoxia. In addition, DNP increased urinary protein excretion, kidney vimentin expression, and infiltration of inflammatory cells. Friederich-Persson et al. thus confirmed that kidney tissue hypoxia, without confounding hyperglycemia or oxidative stress, may be sufficient to initiate the development of nephropathy.

**Figure 1:**
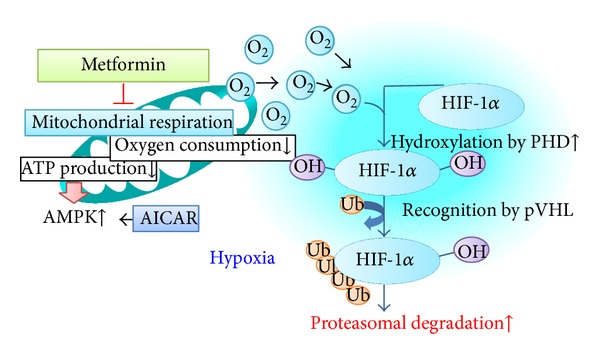
Metformin redistributes the intracellular oxygen. Metformin inhibits oxygen consumption and ATP production by inhibiting mitochondrial complex I. Subsequently, intracellular oxygen redistribution supplies oxygen for prolyl hydroxylase, which promotes the degradation of HIF-1*α* in the proteasome. ATP depletion caused by mitochondrial inhibition activates AMPK, which is a downstream signaling pathway of mitochondrial respiratory chain [21].

We previously showed that the antidiabetes drug metformin suppresses mitochondrial respiratory function, suggesting that the redistribution of intracellular oxygen is involved in the inhibitory effects of metformin on HIF-1*α* expression (Figure 1) [21]. Our results also suggest that metformin inhibits mitochondrial respiration, indicated as the inhibition of oxygen consumption and intracellular ATP levels, which subsequently activates AMPK pathways, suggesting that AMPK*α* is a downstream regulator of the mitochondrial respiratory chain, and metformin-induced AMPK phosphorylation is not related to HIF-1*α* inhibition (Figure 1). Moreover, our preclinical study using type 2 diabetic model Zucker diabetic fatty (ZDF) rats confirmed the renoprotective effects of metformin in vivo. Therefore, the therapeutic target for diabetic kidneys should be to prevent excessive mitochondrial oxygen consumption or to restore renal oxygen availability, not the accompanying oxidative stress or downstream HIF activation.

## 2. Sirtuins, Pseudohypoxia, and NAD^+^-Related HIF

Sirtuins (SIRTs) are NAD-dependent Class III HDACs and their deacetylase activity is controlled by the cellular [NAD^+^]/[NADH] ratio, and age-, hypoxia-, and hyperglycemia-related NAD^+^ deficiency lead to a reduction of SIRTs activity [25]. Previous studies, especially, proposed that SIRT1 has been linked to diabetes [26–28]. SIRT1 mRNA expression may be associated with energy expenditure and insulin sensitivity in the offspring of type 2 diabetic patients [26]. Moreover, a SIRT1 mutation was identified in a family with type 1 diabetes [27, 28].

In 1993, Williamson et al. first referred to pseudohypoxia as an increase in cytosolic NADH/NAD^+^, caused by an increased rate of reduction of NAD^+^ to NADH [29]. They proposed that the tissue lactate/pyruvate ratio is a more reliable parameter of the cytosolic ratio of free NADH/NAD^+^, because the oxidation of NADH to NAD^+^ by LDH is coupled to the reduction of pyruvate to lactate. They also stated that the increased metabolism of glucose via the sorbitol pathway is the most important mechanism for pseudohypoxia [29]. Interestingly, cells with an increased NADH/NAD^+^ ratio attributable to hyperglycemic pseudohypoxia require less severe hypoxia or ischemia to increase NADH/NAD^+^ to the same level caused by hypoxia or ischemia alone. The relationship between pseudohypoxia and hypoxia has not yet been determined. Recent findings indicate that a number of nonhistone substrates, such as HIF-1, are deacetylated by sirtuin family members, expanding the physiological and pathophysiological roles of sirtuins, more than longevity and metabolism.

**Figure 2:**
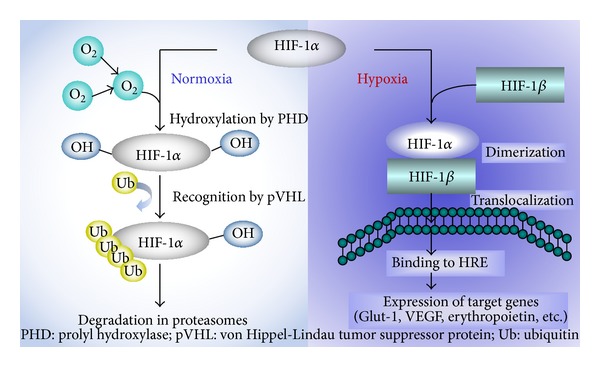
Oxygen regulates HIF-1*α* protein expression. HIF-1 is a heterodimeric transcription factor complex that is composed of an oxygen-regulated HIF-1*α* subunit and a constitutively expressed HIF-1*β* subunit; HIF-1 activity depends on the degradation of HIF-1*α* subunit; the half-life of HIF-1*α* protein on reoxygenation is less than one minutes [31].

HIF-1 is a heterodimeric transcription factor complex composed of an oxygen-regulated HIF-1*α* subunit and a constitutively expressed HIF-1*β* subunit, and HIF-1 activity depends on the degradation of the HIF-1*α* subunit. In normoxia, specific Fe^2+^- and oxoglutarate-dependent prolyl 4-hydroxylases (PHDs) hydroxylate HIF-1*α* at two prolyl sites of the oxygen-dependent domain (ODD). The hydroxylated HIF-1*α* binds to the von Hippel-Lindau (VHL) tumor suppressor protein that is part of an E3 ubiquitin ligase complex targeting HIF-1 for proteasomal degradation [30] (Figure 2).

The expression of HIF-1*α* protein is tightly coupled to the intracellular oxygen concentration, and the half-life of HIF-1*α* protein on reoxygenation is less than 1 min [31]. Accumulating evidence demonstrates that HIF-1 is a target of epigenetic regulation such as the DNA hypermethylation of gene promoters, histone modifications, small interfering RNAs and microRNAs [32]. In light of the inhibitory effects of histone deacetylase inhibitors (HDAIs), HIF function apparently requires deacetylase-dependent transactivation [33].

**Figure 3:**
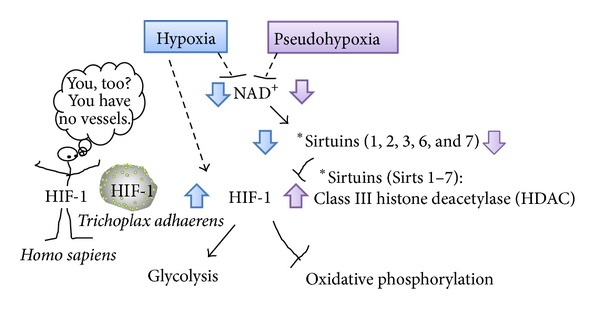
NAD^+^-sirtuins modulate glucose metabolism via HIF-1 in all metazoan species from the simplest animal* Trichoplax adhaerens* to human. Sirtuins (SIRT) consist of seven mammalian proteins, SIRTs 1–7. Some isoforms of sirtuins inhibit HIF-1 activation by deacetylation or noncatalyzing effects [34–38]. Under hypoxia or pseudohypoxia, decreased NAD^+^ levels downregulated SIRT, leading to upregulated HIF-1 activation which shift the glucose metabolism by promoting glycolysis and by inhibiting oxidative phosphorylation.

Interestingly, some isoforms of sirtuins have been known to modulate HIF-1 activity [34–38] (Figure 3). SIRTs consist of seven mammalian proteins, SIRTs 1–7. During hypoxia, decreased NAD^+^ levels downregulated SIRT1, which allowed the acetylation and activation of HIF-1*α* [34]. SIRT1 inactivates HIF-1*α* by binding to HIF-1*α* and deacetylating it and by blocking coactivator p300 recruitment and consequently repressed HIF-1 targeted genes [34]. SIRT2 also regulates the autoacetylation of p300, in vitro and in cells [35], and thus it might modulate HIF-1 activation. Mitochondrial SIRT3 destabilizes HIF-1*α*, which controls glycolytic gene expressions, opposing the reprogramming of cancer cell metabolism, known as the Warburg effect [36]. SIRT6-deficient mice develop normally but die early due to lethal hypoglycemia [37]. As SIRT6 functions as a corepressor of HIF-1*α* and controls the expressions of multiple glycolytic genes, SIRT6-deficient cells exhibit increased HIF-1*α* activity and show increased glucose uptake with an upregulation of glycolysis and diminished mitochondrial respiration [37]. At last, SIRT7 negatively affects HIF-1*α* and HIF-2*α* protein levels by a mechanism that is independent of prolyl hydroxylation and that does not involve proteasomal or lysosomal degradation [38]. Then the mechanism by which SIRT7 regulates HIF activity differs from those of the other sirtuins because of the independence of its deacetylase activity. SIRT7 could regulate HIF through protein-protein interactions, not by enzymatic activity [38].

Although it is due to a complex metabolic scenario of pathological mechanisms, pseudohypoxia might be defined as a paucity of NAD^+^, leading to sirtuins-HIF interaction (Figure 3). Indeed, pseudohypoxia should be considered hyperglycemia-induced metabolic hypoxia, presenting the activation of HIF, a pathophysiological state in which, although oxygen is present, oxygen is less used in mitochondrial respiration by secondary mitochondrial dysfunction, similar to the Warburg effect in cancer cells. Therefore, cells with pseudohypoxia, which has already induced HIF-1, do not need severe hypoxia for inducing HIF-1, and this fraternal hypoxia results in the preference for glycolysis.

The pyruvate/lactate ratio during normoxic conditions in early diabetes may be explained by either increased gluconeogenesis or by glycolysis, not by making an effect on oxidative phosphorylation. In addition, hyperglycemia activates NADPH oxidase (NOX), which critically provides additional NAD^+^ for glycolysis. NOXs are one of the many sources of reactive oxygen species (ROS) [39]. NOX4, which is highly expressed by human vascular endothelial cells, is the predominant form in the kidneys. NOX4 is known to be a novel target for HIF-1 [40] and specifically inhibits mitochondrial complex I [41], leading to mitochondrial dysfunction. Therefore, in diabetic kidney, hyperglycemia and hypoxia may induce glycolysis partly via activation of NOX4 by providing NAD^+^ for fuel and by suppressing mitochondrial oxidative phosphorylation.

## 3. AV Shunting and Polar Vasculosis

Originally, the kidneys are away from hypoxia, because they receive approximately 25% of the cardiac output at only less than 1% of the total body weight. Oxygen delivery to the human kidney is 84 mL/min/100 g, and oxygen consumption in the kidney is 6.8 mL/min/100 g [15]. The renal oxygen delivery thus exceeds the renal oxygen consumption. However, the kidneys normally do not suffer from hyperoxia; rather they are susceptible to hypoxia as shown in DN as well as acute kidney injury (AKI), which is the paradox of oxygen physiology in the kidneys.

One of the reasons for the sensitivity of the kidneys to hypoxia is diffusional arterial-to-venous (AV) oxygen shunting [42, 43]. It is well known that the pressure of oxygen (pO_2_) in renal veins is above those in the efferent arterioles of the outer cortex, proximal tubules, and distal tubules [44]. In addition, the fractional extraction of oxygen falls with increased renal blood flow (RBF), but renal parenchymal pO_2_ remains unchanged [43]. These findings indicate preglomerular AV oxygen shunting by a countercurrent arrangement of mammalian arteries and veins [45] and indicate that AV oxygen shunting contributes to the dynamic regulation of intrarenal oxygenation [43].

**Figure 4:**
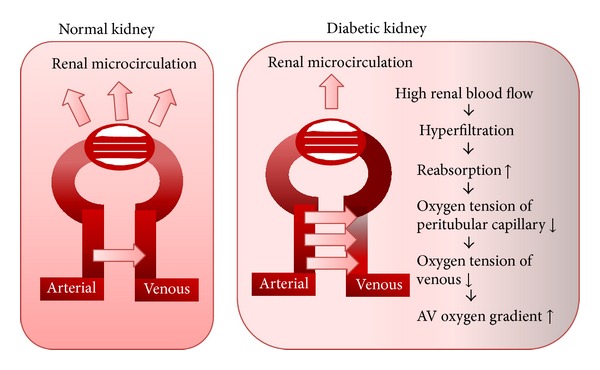
Diabetic kidney exhibits more AV oxygen shunting. Intrarenal oxygen tension is maintained at stable levels by hemodynamic and metabolic interactions of renal blood flow, GFR, oxygen consumption, and arteriovenous (AV) oxygen shunting. Originally, AV oxygen shunting develops as a unique system to rescue kidney from hyperoxia. However, in diabetic kidney, hyperglycemia induces hyperfiltration leading to more AV oxygen gradient which turns in increased AV oxygen shunting. The figure was modified from O'Connor et al. [42].

However, AV oxygen shunting limits the change in oxygen delivery to renal tissue and stabilizes tissue pO_2_ when arterial pO_2_ changes but renders renal parenchyma susceptible to hypoxia when oxygen delivery falls or parenchymal oxygen consumption increases [46]. In physiologically normal conditions, this AV oxygen shunting preserves the antioxidant defense mechanisms in the kidneys from hyperoxia-induced reactive oxygen superoxide production, by which the kidneys could cope with superfluous oxygen as like in the lung with its pO_2_ close to atmospheric pO_2_ (159 mmHg) [45]. The kidneys are second to the heart in terms of oxygen consumption, especially for sodium transport. For the first time in the evolution of humans, the kidneys are experiencing exceeded metabolic demand, because of hypernutrition. Since hyperglycemia increases the renal blood flow and then the tubular reabsorption of glucose and erythrocytes (which need oxygen consumption), leading to decreased oxygen tension of peritubular capillaries, the increased oxygen gradient between arterioles and veins augments the AV oxygen shunting, promoting renal parenchymal hypoxia (Figure 4). Then, the AV oxygen shunting preferentially promotes hypoxia-induced injury in diabetic kidney rather than an escape from hyperoxia.

Interestingly, retinal venous oxygen saturation is increased in diabetic patients with the severity of the diabetic retinopathy, although no significant differences in the retinal arterial oxygen saturation were observed between controls and patients with diabetic retinopathy at any stage, suggesting the involvement of the occlusions and obliterations in the retinal capillary bed, leading to the formation of AV shunting vessels [47]. Similarly, physiological preglomerular AV shunting might make an important contribution to the sequential decline of the glomerular filtration rate (GFR) caused by the glomerular hypertension in DN.

Although cortical tissue oxygenation is independent of medullary blood flow and oxygenation, medullary tissue pO_2_ appears to be dependent on the levels of both cortical and medullary perfusion [48], which seem to have equal effects on renal perfusion pressure (RPP). Kidneys have an autoregulation system in which the total renal blood flow (RBF) is autoregulated between the RPP values of 90 and 200 mmHg. However, the blood flow to the medulla is not autoregulated [49]. Three decades ago, Casellas and Mimran demonstrated that one-tenth of all juxtamedullary glomeruli have anatomical shunts with preglomerular vessels to postglomerular vasa recta capillaries [50]. Since these shunts arise proximally to the afferent arterioles, these would not be subject to tubuloglomerular feedback and autoregulation. Although the medullas of the kidneys receive less than 10% of the total RBF, the medullary blood flow responding to an elevation of systemic arterial pressure might contribute to the high oxygen tension in the outer stripe of the outer medulla, as in the superficial cortex [51].

Intriguingly, there is another specific vascular architecture involved in the pathogenesis of diabetic kidneys, that is, polar vasculosis [52–54]. Like the new vessel formation in diabetic retinopathy, neovascularization at the glomerular vascular pole was described in diabetic nephropathy [52–54]. Min and Yamanaka demonstrated that 72% of 73 autopsy cases and 21 biopsy cases of diabetic glomerulonephropathy had multiple small vessels around the polar vascular pole which connect at a point after two or three branches from the afferent arteriole, and their opposite ends outside the glomerulus anastomose to peritubular capillaries [53]. Those authors proposed that these vessels probably compensate for the decreased function of efferent arterioles because of sclerosis, which is a characteristic finding in diabetic nephropathy [55]. Although this vascular abnormality was specific to diabetic kidneys, the pathological role of polar vasculosis remains obscure. The neovascularization might lower the intraglomerular pressure by shunting afferent and efferent arteries, and it may reveal a compensatory response for glomerular hypertension. However, the diameters of these abnormal arterioles are so small that they might be affected by arteriosclerosis and are obstructed, leading to the progression of renal dysfunction.

## 4. Roles of HIF in Diabetic Kidneys

**Figure 5:**
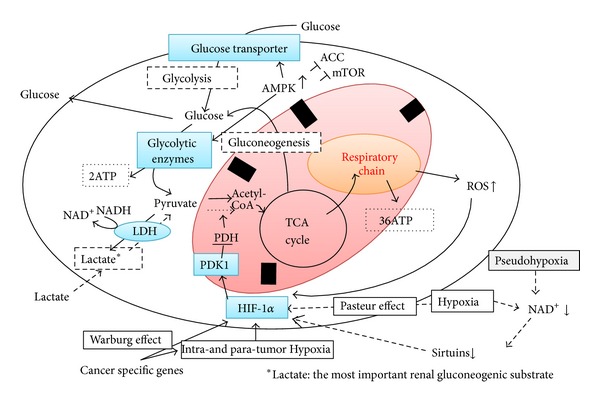
The essential role of HIF-1 is to switch the glucose metabolism from oxidative phosphorylation to glycolysis under hypoxic (Pasteur effect) or normoxic (pseudohypoxia or Warburg effect) conditions. Glycolysis: glucose + 2P_*i*_ + 2ADP + 2NAD^+^→ 2pyruvate + 2ATP + 2NADH + 2H_2_O, 2pyruvate + 2NADH → 2lactate + 2NAD^+^. Aerobic respiration: glucose + 6O_2_ + 36P_*i*_ + 36ADP → 6CO_2_ + 36ATP + 42H_2_O.

HIF-1 was discovered in an investigation of the regulation of human erythropoietin gene, which encodes erythropoietin [56], and it was later shown to regulate VEGF expression [57]. These earlier studies indicated the pivotal roles of HIF-1 in hematogenesis and angiogenesis. All metazoan species including the simplest animal* Trichoplax adhaerens* which totally has no specialized systems for oxygen delivery, such as blood cells or vessels, possess HIF-1 [58]. HIF targets in* T. adhaerens* include glycolytic and metabolic enzymes, suggesting a primary role for HIF in the regulation of oxygen consumption as an adaptation of basal multicellular animals to fluctuating oxygen levels. Of note,* taPDK* is among the strongest hypoxically induced genes in* T. adhaerens*, and human pyruvate dehydrogenase (PDH) kinase 1 (PDK1) is also an HIF target gene [58]. PDK1 inhibits the PDH-catalyzed conversion of pyruvate to acetyl-coenzyme A (acetyl-CoA) for the entry into mitochondrial tricarboxylic acid (TCA) cycle (Figure 5). Therefore, HIF in* T. adhaerens* also switches from oxidative phosphorylation to glycolysis. Since HIF originally functions as master regulator of oxygen homeostasis in all metazoan species, HIF also regulates glucose and energy metabolism to adapt to reduced oxygen availability in hypoxia-susceptible diabetic kidneys. Under a sustained hypoxic condition, HIF subsequently remodels the hemodynamics by producing blood cells and by inducing new vessels, via, respectively, targeted erythropoietin and VEGF. Even after this new HIF-induced microenvironmental reprogramming, hypoxia still exists, and then HIF would eliminate these hypoxic lesions as seen in wounds or infections distant from healthy tissues by surrounding fibrotic capsules.

Using HIF-1*α* knockout mice, Higgins et al. demonstrated that HIF-1*α* enhanced the epithelial-to-mesenchymal transition (EMT) in vitro and that the genetic ablation of renal epithelial HIF-1*α* inhibited the development of tubulointerstitial fibrosis [10]. Sun et al. further provided a novel explanation for the EMT of hypoxic renal tubular cells through the upregulation of Twist induced by HIF-1*α* [59]. Kimura et al. showed that the stable expression of HIF-1*α* in tubular epithelial cells promotes interstitial fibrosis in knockout mice with VHL tumor suppressor, which acts as an ubiquitin ligase to promote the proteolysis of HIF-1*α* [60]. If HIF can play a renoprotective role in diabetic kidneys, HIF expression would not be observed in diabetic kidneys, because HIF rescues the kidneys from hypoxia, and its signal will vanish within a few minutes. Therefore, when HIF is observed in diabetic kidneys, it means that HIF cannot conquer persistent hypoxia. The long-standing expression of HIF induces deleterious phenomena such as renal fibrosis. Two recent studies also clarified that the continuous activation of HIF-1 or HIF-1-targeted molecules contributed to chronic kidney diseases [61, 62]. Wang et al. showed that silencing of HIF-1*α* gene attenuates chronic ischemic renal injury in two-kidney one-clip rats [61]. Gobe et al. demonstrated that recombinant human erythropoietin protected the tubular epithelium from apoptosis but stimulated EMT, and the supraphysiological dose needed for renoprotection contributed to fibrogenesis and stimulated chronic kidney disease in the long term [62]. This adverse effects of erythropoietin remind us of the potential disadvantage of the HIF stabilizer, FG-4592, which is an oral compound in Phase III trials for the treatment of anemia in ESRD patients. FG-4592, an inhibitor of HIF prolyl hydroxylase (HIF-PH), prevents HIF degradation and stimulates the erythropoietin production [63]. The therapeutic short activation of HIF may be protective for kidneys by switching from oxidative phosphorylation to glycolysis, accompanied by decreased oxygen consumption, suppressed ROS production, and minimum ATP production. On the contrary, the chronic activation of HIF does not seem to have potential in the treatment of diabetic kidneys, because HIF would consistently mediate hypoxia-caused cell injury via the HIF-downstream molecules. In addition, chronically enhanced HIF expression implies abnormal cell metabolism as is common in cancer. We cannot exclude the possibility that HIF promotes carcinogenesis. Further studies are required to clarify the mechanisms underlying diabetic hypoxic kidneys and to understand the over- and sustained expressions of HIF-promoted cell metabolism. Furthermore, it has been recently shown that the therapeutic target for diabetic kidneys could be simply hypoxia, not HIF. This point was brought into focus by the findings of Friederich-Persson and colleagues [24].

## 5. Why Hypoxia in Diabetic Kidneys?

As described above, the kidneys are very sensitive to hypoxia, because of the energy demand for its function, and this is the reason why hypoxia contributes to the glucose metabolism. The kidney is unique organ that uptakes, uses, and produces glucose. Kidneys need oxygen for the reabsorption of filtrated glucose and electrolytes, and they produce ATP via oxidative phosphorylation and glycolysis. Under hypoxic conditions, to produce ATP to maintain kidney function, the kidneys use anaerobic fermentation by Pasteur effects. Even in normoxia, hyperglycemia causes an increased glucose influx along with activated glycolysis, which leads to other glucose submetabolic pathways such as polyol pathways. Therefore, hyperglycemia promotes glycolysis and other metabolic pathways rather than mitochondrial oxidative phosphorylation in normoxia, which resembles the Warburg effect that plays an important role in cancer metabolism.

HIF plays a key role in the glycolytic switch by the regulation of genes encoding most glycolytic transporters and enzymes and then enhancing renal medullary glycolysis. By using hyperpolarized [1-^13^C] pyruvate MRI, Laustsen et al. demonstrated that reduced oxygen availability in streptozotocin diabetic rat kidney altered the energy metabolism by increasing lactate and alanine formation [64]. In addition to diabetes-induced pseudohypoxia, reduced oxygen content in inspired air increased the pyruvate-to-lactate and pyruvate-to-alanine formation accompanied by a reduced NAD^+^/NADH ratio. Both increased the mRNA expression of lactate dehydrogenase A (LDHA) which metabolites pyruvate to lactate and alanine aminotransferase (ALT) which converts pyruvate to alanine, suggesting that these two pathways may contribute to reducing substrate flux into the TCA cycle and limiting oxidative phosphorylation [64]. The similar pyruvate depletion in kidney is known during AKI in mice [65]. Although lactate levels in AKI mice kidneys fell below control levels, the pyruvate levels remained depressed, because AKI increases gluconeogenesis, which results in pyruvate consumption [65].

The kidney is known to be a significant gluconeogenic organ, and in overnight-fasted normal humans, proximal tubule gluconeogenesis is responsible for approximately 40% of whole-body gluconeogenesis [66]. Renal and hepatic glucose release in human diabetic subjects with type 2 diabetes are increased [67]. A study of ZDF rats also showed that renal gluconeogenesis is upregulated accompanied by an increase in the activities and mRNA levels of the key gluconeogenic enzymes [68].

**Figure 6:**
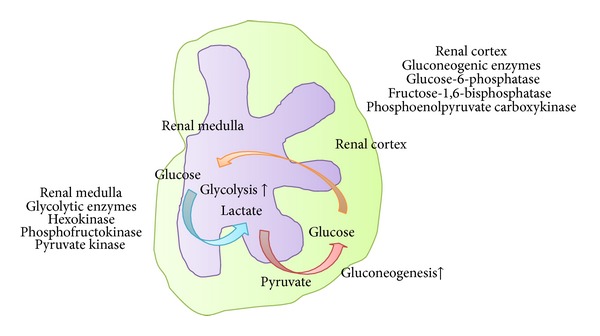
Glycolysis and gluconeogenesis in the kidney. A lactate shuttle may operate in the kidney between medulla and cortex as a main source of energy to sustain renal physiology. In diabetic kidneys, HIF may augment the medullary glycolysis rather than oxidative phosphorylation, leading to the production of lactate, which could be taken up by the cortical proximal tubules for gluconeogenesis. Then, renal glucose release in diabetic kidney is increased.

The proximal tubule reabsorbs glucose and synthesizes glucose, known as “gluconeogenesis,” but does not metabolize glucose. The renal medulla utilizes glucose and generates lactate for its glycolysis. Simultaneously, the cortical proximal tubules take up the lactate released by the medulla, use it for oxidative phosphorylation, and then generate and release glucose for the energy production by the medullary glycolysis (Figure 6). A similar lactate shuttle may operate in the brain between astrocytes and neurons as a main source of energy to sustain neuronal physiology [69]. In diabetic kidneys, HIF may augment the medullary glycolysis rather than oxidative phosphorylation, leading to the production of lactate, which could be taken up by the cortical proximal tubules for gluconeogenesis. HIF thus could remodel the energy production system for diabetic kidneys under hypoxia. In contrast, in AKI, HIF fails to work, which might result in deficits of medullary glycolysis and subsequent cortical gluconeogenesis, because the oxygen-sensitive medulla does not suffer severe hypoxia by relative lack of oxygen consumption for sodium reabsorption, due to a marked decrease in GFR [70].

In conclusion, if human beings do not develop diabetes mellitus, under a blood supply that is one-fourth of the cardiac output, AV oxygen shunting and high capacity for production of energy by rich mitochondria could protect the kidneys from hyperoxia-induced ROS. Diabetes mellitus increases the renal blood flow, glomerular filtration, sodium absorption, need for ATP production, and oxygen consumption. Diabetes mellitus thus induces hypoxia in the kidneys. The systems that protect the kidneys from hyperoxia ironically promote the susceptibility of diabetic kidneys to hypoxic injury.

## References

[1] Orchard TJ, Secrest AM, Miller RG, Costacou T (2010). In the absence of renal disease, 20 year mortality risk in type 1 diabetes is comparable to that of the general population: a report from the Pittsburgh Epidemiology of Diabetes Complications Study. *Diabetologia*.

[2] Afkarian M, Sachs MC, Kestenbaum B (2013). Kidney disease and increased mortality risk in type 2 diabetes. *Journal of the American Society of Nephrology*.

[3] Nangaku M (2006). Chronic hypoxia and tubulointerstitial injury: a final common pathway to end-stage renal failure. *Journal of the American Society of Nephrology*.

[4] Haase VH (2006). The VHL/HIF oxygen-sensing pathway and its relevance to kidney disease. *Kidney International*.

[5] Eckardt K-U, Bernhardt W, Willam C, Wiesener M (2007). Hypoxia-inducible transcription factors and their role in renal disease. *Seminars in Nephrology*.

[6] Singh DK, Winocour P, Farrington K (2008). Mechanisms of disease: the hypoxic tubular hypothesis of diabetic nephropathy. *Nature Clinical Practice Nephrology*.

[7] Ries M, Basseau F, Tyndal B (2003). Renal diffusion and BOLD MRI in experimental diabetic nephropathy. *Journal of Magnetic Resonance Imaging*.

[8] Palm F, Hansell P, Ronquist G, Waldenström A, Liss P, Carlsson P-O (2004). Polyol-pathway-dependent disturbances in renal medullary metabolism in experimental insulin-deficient diabetes mellitus in rats. *Diabetologia*.

[9] Rosenberger C, Khamaisi M, Abassi Z (2008). Adaptation to hypoxia in the diabetic rat kidney. *Kidney International*.

[10] Higgins DF, Kimura K, Bernhardt WM (2007). Hypoxia promotes fibrogenesis in vivo via HIF-1 stimulation of epithelial-to-mesenchymal transition. *The Journal of Clinical Investigation*.

[11] Thelwall PE, Taylor R, Marshall SM (2011). Non-invasive investigation of kidney disease in type 1 diabetes by magnetic resonance imaging. *Diabetologia*.

[12] Pruijm M, Hofmann L, Zanchi A (2013). Blockade of the renin-angiotensin system and renal tissue oxygenation as measured with BOLD-MRI in patients with type 2 diabetes. *Diabetes Research and Clinical Practice*.

[13] Inoue T, Kozawa E, Okada H (2011). Noninvasive evaluation of kidney hypoxia and fibrosis using magnetic resonance imaging. *Journal of the American Society of Nephrology*.

[14] Thaysen JH, Lassen NA, Munck O (1961). Sodium transport and oxygen consumption in the mammalian kidney. *Nature*.

[15] McDonuogh AA, Thomson SC, Brebbber B, Rector F (2011). Metabolic basis of solute transport. *The Kidney: Physiology and Pathophysiology*.

[16] Jerums G, Premaratne E, Panagiotopoulos S, MacIsaac RJ (2010). The clinical significance of hyperfiltration in diabetes. *Diabetologia*.

[17] Gullans SR, Harris SI, Mandel LJ (1984). Glucose-dependent respiration in suspensions of rabbit cortical tubules. *Journal of Membrane Biology*.

[18] Brezis M, Agmon Y, Epstein FH (1994). Determinants of intrarenal oxygenation: I. Effects of diuretics. *American Journal of Physiology—Renal Fluid and Electrolyte Physiology*.

[19] Epstein FH, Prasad P (2000). Effects of furosemide on medullary oxygenation in younger and older subjects. *Kidney International*.

[20] Cherney DZI, Perkins BA, Soleymanlou N (2014). Renal hemodynamic effect of sodium-glucose cotransporter 2 inhibition in patients with type 1 diabetes mellitus. *Circulation*.

[21] Takiyama Y, Harumi T, Watanabe J (2011). Tubular injury in a rat model of type 2 diabetes is prevented by metformin: a possible role of HIF-1*α* expression and oxygen metabolism. *Diabetes*.

[22] Katavetin P, Miyata T, Inagi R (2006). High glucose blunts vascular endothelial growth factor response to hypoxia via the oxidative stress-regulated hypoxia-inducible factor/hypoxia-responsible element pathway. *Journal of the American Society of Nephrology*.

[23] Brownlee M (2005). The pathobiology of diabetic complications: a unifying mechanism. *Diabetes*.

[24] Friederich-Persson M, Thörn E, Hansell P, Nangaku M, Levin M, Palm F (2013). Kidney hypoxia, attributable to increased oxygen consumption, induces nephropathy independently of hyperglycemia and oxidative stress. *Hypertension*.

[25] Imai S-I, Armstrong CM, Kaeberlein M, Guarente L (2000). Transcriptional silencing and longevity protein Sir2 is an NAD-dependent histone deacetylase. *Nature*.

[26] Rutanen J, Yaluri N, Modi S (2010). SIRT1 mRNA expression may be associated with energy expenditure and insulin sensitivity. *Diabetes*.

[27] Chong ZZ (2013). Diabetes: implications of a novel point mutation of SIRT1 in T1DM. *Nature Reviews Endocrinology*.

[28] Biason-Lauber A, Böni-Schnetzler M, Hubbard BP (2013). Identification of a SIRT1 mutation in a family with type 1 diabetes. *Cell Metabolism*.

[29] Williamson JR, Chang K, Frangos M (1993). Hyperglycemic pseudohypoxia and diabetic complications. *Diabetes*.

[30] Semenza GL (2011). Mechanisms of disease: oxygen sensing, homeostasis, and disease. *The New England Journal of Medicine*.

[31] Yu AY, Frid MG, Shimoda LA, Wiener CM, Stenmark K, Semenza GL (1998). Temporal, spatial, and oxygen-regulated expression of hypoxia-inducible factor-1 in the lung. *American Journal of Physiology—Lung Cellular and Molecular Physiology*.

[32] Nguyen MP, Lee S, Lee YM (2013). Epigenetic regulation of hypoxia inducible factor in diseases and therapeutics. *Archives of Pharmacal Research*.

[33] Chen S, Sang N (2011). Histone deacetylase inhibitors: the epigenetic therapeutics that repress hypoxia-inducible factors. *Journal of Biomedicine and Biotechnology*.

[34] Lim J-H, Lee Y-M, Chun Y-S, Chen J, Kim J-E, Park J-W (2010). Sirtuin 1 modulates cellular responses to hypoxia by deacetylating hypoxia-inducible factor 1alpha. *Molecular Cell*.

[35] Black JC, Mosley A, Kitada T, Washburn M, Carey M (2008). The SIRT2 deacetylase regulates autoacetylation of p300. *Molecular Cell*.

[36] Finley LWS, Carracedo A, Lee J (2011). SIRT3 opposes reprogramming of cancer cell metabolism through HIF1*α* destabilization. *Cancer Cell*.

[37] Zhong L, D’Urso A, Toiber D (2010). The histone deacetylase Sirt6 regulates glucose homeostasis via Hif1*α*. *Cell*.

[38] Hubbi ME, Hu H, Gilkes DM, Semenza GL (2013). Sirtuin-7 inhibits the activity of hypoxia-inducible factors. *The Journal of Biological Chemistry*.

[39] Sedeek M, Nasrallah R, Touyz RM, Hébert RL (2013). NADPH oxidases, reactive oxygen species, and the kidney: friend and foe. *Journal of the American Society of Nephrology*.

[40] Diebold I, Petry A, Hess J, Görlach A (2010). The NADPH oxidase subunit NOX4 is a new target gene of the hypoxia-inducible factor-1. *Molecular Biology of the Cell*.

[41] Kozieł R, Pircher H, Kratochwil M (2013). Mitochondrial respiratory chain complex i is inactivated by NADPH oxidase Nox4. *Biochemical Journal*.

[42] O’Connor PM, Anderson WP, Kett MM, Evans RG (2006). Renal preglomerular arterial-venous O_2_ shunting is a structural anti-oxidant defence mechanism of the renal cortex. *Clinical and Experimental Pharmacology and Physiology*.

[43] Leong C-L, Anderson WP, O’Connor PM, Evans RG (2007). Evidence that renal arterial-venous oxygen shunting contributes to dynamic regulation of renal oxygenation. *American Journal of Physiology—Renal Physiology*.

[44] Welch WJ, Baumgärtl H, Lübbers D, Wilcox CS (2001). Nephron pO_2_ and renal oxygen usage in the hypertensive rat kidney. *Kidney International*.

[45] O’Connor PM, Evans RG (2010). Structural antioxidant defense mechanisms in the mammalian and nonmammalian kidney: different solutions to the same problem?. *American Journal of Physiology—Regulatory Integrative and Comparative Physiology*.

[46] Gardiner BS, Smith DW, O’Connor PM, Evans RG (2011). A mathematical model of diffusional shunting of oxygen from arteries to veins in the kidney. *American Journal of Physiology—Renal Physiology*.

[47] Hammer M, Vilser W, Riemer T (2009). Diabetic patients with retinopathy show increased retinal venous oxygen saturation. *Graefe’s Archive for Clinical and Experimental Ophthalmology*.

[48] O’Connor PM, Kett MM, Anderson WP, Evans RG (2006). Renal medullary tissue oxygenation is dependent on both cortical and medullary blood flow. *American Journal of Physiology—Renal Physiology*.

[49] Mattson DL, Lu S, Roman RJ, Cowley AW (1993). Relationship between renal perfusion pressure and blood flow in different regions of the kidney. *American Journal of Physiology—Regulatory Integrative and Comparative Physiology*.

[50] Casellas D, Mimran A (1981). Shunting in renal microvasculature of the rat: a scanning electron microscopic study of corrosion casts. *Anatomical Record*.

[51] Palm F, Hansell P, Ronquist G, Waldenström A, Liss P, Carlsson P-O (2004). Polyol-pathway-dependent disturbances in renal medullary metabolism in experimental insulin-deficient diabetes mellitus in rats. *Diabetologia*.

[52] Meyer C, Woerle HJ, Dostou JM, Welle SL, Gerich JE (2004). Abnormal renal, hepatic, and muscle glucose metabolism following glucose ingestion in type 2 diabetes. *American Journal of Physiology—Endocrinology and Metabolism*.

[53] Min W, Yamanaka N (1993). Three-dimensional analysis of increased vasculature around the glomerular vascular pole in diabetic nephropathy. *Virchows Archiv A: Pathological Anatomy and Histopathology*.

[54] Østerby R, Asplund J, Bangstad H-J (1999). Neovascularization at the vascular pole region in diabetic glomerulopathy. *Nephrology Dialysis Transplantation*.

[55] Bell ET (1953). Renal vascular disease in diabetes mellitus. *Diabetes*.

[56] Semenza GL, Wang GL (1992). A nuclear factor induced by hypoxia via de novo protein synthesis binds to the human erythropoietin gene enhancer at a site required for transcriptional activation. *Molecular and Cellular Biology*.

[57] Forsythe JA, Jiang B-H, Iyer NV (1996). Activation of vascular endothelial growth factor gene transcription by hypoxia-inducible factor 1. *Molecular and Cellular Biology*.

[58] Loenarz C, Coleman ML, Boleininger A (2011). The hypoxia-inducible transcription factor pathway regulates oxygen sensing in the simplest animal, Trichoplax adhaerens. *EMBO Reports*.

[59] Sun S, Ning X, Zhang Y (2009). Hypoxia-inducible factor-1*α* induces Twist expression in tubular epithelial cells subjected to hypoxia, leading to epithelial-to-mesenchymal transition. *Kidney International*.

[60] Kimura K, Iwano M, Higgins DF (2008). Stable expression of HIF-1*α* in tubular epithelial cells promotes interstitial fibrosis. *American Journal of Physiology—Renal Physiology*.

[61] Wang Z, Zhu Q, Li PL (2014). Silencing of hypoxia-inducible factor-1*α* gene attenuates chronic ischemic renal injury in two-kidney, one-clip rats. *American Journal of Physiology—Renal Physiology*.

[62] Gobe GC, Bennett NC, West M (2014). Increased progression to kidney fibrosis after erythropoietin is used as a treatment for acute kidney injury. *American Journal of Physiology—Renal Physiology*.

[63] Bunn HF (2007). New agents that stimulate erythropoiesis. *Blood*.

[64] Laustsen C, Lycke S, Palm F (2013). High altitude may alter oxygen availability and renal metabolism in diabetics as measured by hyperpolarized [1-^13^C]pyruvate magnetic resonance imaging. *Kidney International*.

[65] Zager RA, Johnson AC, Becker K (2014). Renal cortical pyruvate depletion during AKI. *Journal of the American Society of Nephrology*.

[66] Gerich JE, Meyer C, Woerle HJ, Stumvoll M (2001). Renal gluconeogenesis: its importance in human glucose homeostasis. *Diabetes Care*.

[67] Meyer C, Stumvoll M, Nadkarni V, Dostou J, Mitrakou A, Gerich J (1998). Abnormal renal and hepatic glucose metabolism in type 2 diabetes mellitus. *The Journal of Clinical Investigation*.

[68] Eid A, Bodin S, Ferrier B (2006). Intrinsic gluconeogenesis is enhanced in renal proximal tubules of Zucker diabetic fatty rats. *Journal of the American Society of Nephrology*.

[69] Benarroch EE (2014). Brain glucose transporters: implications for neurologic disease. *Neurology*.

[70] Abdelkader A, Ho J, Ow CP (2014). Renal oxygenation in acute renal ischemia-reperfusion injury. *American Journal of Physiology—Renal Physiology*.

